# Incidence and Clinical Outcomes of Postoperative Acute Kidney Injury in Relation to Etiological Factors and Surgical Procedures

**DOI:** 10.7759/cureus.89155

**Published:** 2025-07-31

**Authors:** Muhammad Tahir, Haq Nawaz, Abdullah Iqbal, Humayun Safdar, Mahnoor Naeem, Adeel Ahmed, Muhammad Irfan Jamil, Faryal Ghani, Muhammad Ayoob Memon, Maha Afzal

**Affiliations:** 1 General Surgery, Naas General Hospital, Naas, IRL; 2 General Surgery, Sahiwal Teaching Hospital, Sahiwal, PAK; 3 General Surgery, Hayatabad Medical Complex Peshawar, Peshawar, PAK; 4 General Surgery, Lahore General Hospital, Lahore, PAK; 5 Medicine, Mayo Hospital Lahore, Lahore, PAK; 6 Anesthesiology and Critical Care, Lahore General Hospital, Lahore, PAK; 7 Nephrology, Lahore General Hospital, Lahore, PAK; 8 Cardiology, Glenfield Hospital, Leicester, GBR; 9 General Surgery, Quaid-e-Azam Medical College, Bahawalpur, PAK; 10 Internal Medicine, Jinnah Sindh Medical University, Karachi, PAK; 11 Internal Medicine, Fatima Jinnah Medical University, Lahore, PAK

**Keywords:** intensive care unit, postoperative acute kidney injury, renal outcomes, renal replacement therapy (rrt), risk factors

## Abstract

Background and objective: Postoperative acute kidney injury (AKI) is a frequent yet preventable complication linked with increased morbidity, mortality, and healthcare burden. Multiple perioperative risk factors contribute to its development. This study aimed to determine the incidence, clinical outcomes, and independent predictors of postoperative AKI across various surgical procedures.

Methods: This cross-sectional observational study was conducted at the Department of Surgery at a tertiary care hospital between November 2023 and July 2024. A total of 175 adult patients undergoing general surgical procedures under regional or general anesthesia were enrolled using non-probability consecutive sampling. Patients with chronic kidney disease, end-stage renal disease, or pre-existing AKI were excluded. Clinical, laboratory, and intraoperative variables were recorded using a standardized proforma. Data analysis was performed using IBM SPSS Statistics for Windows, Version 26 (Released 2019; IBM Corp., Armonk, New York, United States), with multivariable logistic regression applied to identify independent predictors of postoperative AKI.

Results: Out of 175 patients, postoperative AKI occurred in 33 individuals (18.9%), predominantly Stage 1 (63.64%). Patients with AKI were older (61.85 ± 8.51 vs. 56.23 ± 11.33 years, p = 0.008) and had higher baseline serum creatinine (1.12 ± 0.25 vs. 0.99 ± 0.15 mg/dL, p < 0.001). Significant associations with AKI included a medical history of hypertension (75.8%, OR: 6.52, p < 0.001), diabetes (45.5%, OR: 3.11, p = 0.004), ischemic heart disease (24.2%, OR: 3.47, p = 0.010), contrast exposure (24.2%, OR: 5.36, p = 0.001), and nephrotoxic drugs (48.5%, OR: 5.74, p < 0.001). ICU admission (45.5%), dialysis (18.2%), and in-hospital mortality (18.2%) were significantly higher in AKI cases (all p < 0.05).

Conclusion: Postoperative AKI was significantly linked with adverse outcomes and identifiable clinical and operative risk factors. Focused preventive strategies and early intraoperative risk mitigation may reduce its incidence and improve surgical safety in high-risk patients.

## Introduction

Postoperative acute kidney injury (AKI) is recognized as a significant complication in surgical practice, defined by a rapid decrease in renal function that may arise within hours to days after an operation [1]. According to the Kidney Disease: Improving Global Outcomes (KDIGO) guidelines, AKI is diagnosed by an increase in serum creatinine of at least 0.3 mg/dL within 48 hours, or to 1.5 times or more the baseline value within seven days, or a urine output less than 0.5 mL/kg/h for six hours [2]. Globally, more than 230 million major surgeries are performed each year, with the incidence of postoperative AKI varying from 0.8% to over 30%, depending on the procedure type and patient profile [3]. Even in general surgical patients, AKI can prolong hospital stay, increase the need for intensive care, and substantially elevate the risk of morbidity and mortality, yet it frequently remains underdiagnosed in routine clinical settings [4].

The development of postoperative AKI involves a complex combination of patient and surgical factors. Intraoperative hypotension, significant blood loss, and fluid shifts can reduce renal perfusion, leading to ischemic injury and, with subsequent reperfusion, oxidative stress and inflammation in the kidney [5]. Exposure to nephrotoxic agents further increases the risk, particularly in major or emergency abdominal surgeries where physiological stress is substantial [6]. The presence of comorbidities diminishes renal reserve, making these patients more susceptible to injury. Additionally, prolonged surgical duration, need for vasopressors or transfusions, and severe systemic inflammatory responses all contribute to the pathogenesis and severity of postoperative AKI [7,8].

Despite its clinical importance, the burden of postoperative AKI remains underexplored in general surgical populations [9]. Most available data focus on cardiac or critical care cohorts, where AKI incidence ranges from 8.5% to 65% depending on procedure complexity and patient risk profile [10]. In contrast, studies in general surgery report a broader and inconsistent incidence range of 0.8% to 39.3%, influenced by heterogeneity in AKI definitions, perioperative monitoring, and inclusion criteria [11]. In a prospective multicenter study, Zarbock et al. documented an overall AKI rate of 18.4% across non-cardiac surgeries [12]. Many previous studies lack standardization, particularly in applying KDIGO criteria or monitoring both serum creatinine and urine output [13,14].

Despite growing awareness of postoperative complications, the burden and trajectory of AKI following general surgical procedures remain insufficiently characterized. Most prior investigations have focused on high-risk or intensive care populations, often overlooking patients undergoing routine operations where preoperative status and intraoperative dynamics may still contribute significantly to renal dysfunction [15,16]. Furthermore, inconsistencies in diagnostic application and limited stratification by surgical type have restricted generalizability. This study aims to assess the incidence, associated risk factors, and clinical outcomes of postoperative AKI in patients undergoing general surgical procedures.

## Materials and methods

This cross-sectional observational study was executed at the Department of Surgery, Lahore General Hospital, Lahore from November 2023 to July 2024, following authorization from the Institutional Ethical Review Board (No. 203/11/2023). A non-probability consecutive sampling was used to enroll patients. A sample size of 175 was calculated based on a hypothesized frequency of postoperative AKI of 20.28%, with an absolute precision of 5% and a 90% confidence level [11].

Adult patients aged 18 years or older of either gender, American Society of Anesthesiologists (ASA) status I to IV, undergoing general surgical procedures under regional or general anesthesia with available preoperative serum creatinine within seven days, and who provided written informed consent were included. Patients were excluded if they had end-stage renal disease on dialysis, chronic kidney disease, pre-existing AKI based on KDIGO criteria, or if they died intraoperatively or within 48 hours postoperatively. Day-care or ambulatory surgery cases without postoperative monitoring were also excluded.

Before enrollment, written informed consent was obtained from each individual. Baseline demographic and clinical variables, including age, gender, body mass index, and comorbidities such as hypertension, diabetes mellitus, and ischemic heart disease, were documented. Preoperative laboratory parameters included serum creatinine, along with hemoglobin levels. Exposure to potential nephrotoxic agents such as contrast media administered for radiological procedures, aminoglycosides or nonsteroidal anti-inflammatory drugs was noted from medical history and prescription records. For contrast media, exposure was classified in a binary manner (exposed vs. not exposed) based on the presence of any documented administration of intravenous contrast for imaging studies within seven days prior to surgery. To minimize observer and misclassification bias, a standardized data collection form was used, and all laboratory measurements were obtained from the hospital’s central lab using consistent protocols.

Perioperative variables such as anesthesia type (regional or general), ASA physical status, duration of surgery (classified as <2 hours or >2 hours), estimated intraoperative blood loss (<300 mL or >300 mL), and whether a blood transfusion was administered during or immediately following the procedure were recorded for each patient. The type of general surgical procedure was noted and categorized into laparotomy, intestinal, hepatobiliary, pancreatic, or minor surgeries based on operative records.

The primary outcome was the development of postoperative AKI, assessed according to KDIGO criteria [2]. AKI was defined as an increase in serum creatinine of ≥0.3 mg/dL within 48 hours, or to ≥1.5 times preoperative baseline value within seven days. Severity of AKI was staged (Stage 1, 2, or 3) in accordance with the extent of creatinine elevation. Secondary outcomes were monitored during the in-hospital stay and up to 30 days post-surgery or until discharge, whichever occurred earlier. These included renal recovery status (complete, partial, or no recovery), need for renal replacement therapy (RRT), admission to the ICU, and in-hospital mortality. Recovery status was evaluated using serial serum creatinine measurements and clinical progress. Complete recovery was defined as return of kidney function to baseline; partial recovery as improvement but not return to baseline; and no recovery as no improvement or continued need for dialysis at discharge.

All statistical analyses were executed using IBM SPSS Statistics for Windows, Version 26 (Released 2019; IBM Corp., Armonk, New York, United States). Quantitative variables were expressed as mean values with standard deviations and compared using independent t-tests. Categorical data were presented as numbers and percentages, with group comparisons conducted using the chi-square test. For continuous variables, effect estimates were calculated as mean differences along with 95% CI, while for categorical variables, OR with corresponding 95% CI was derived. Multivariate logistic regression was utilized to identify independent predictors associated with the onset of postoperative AKI. A p-value below 0.05 was considered statistically significant.

## Results

**Figure 1:**
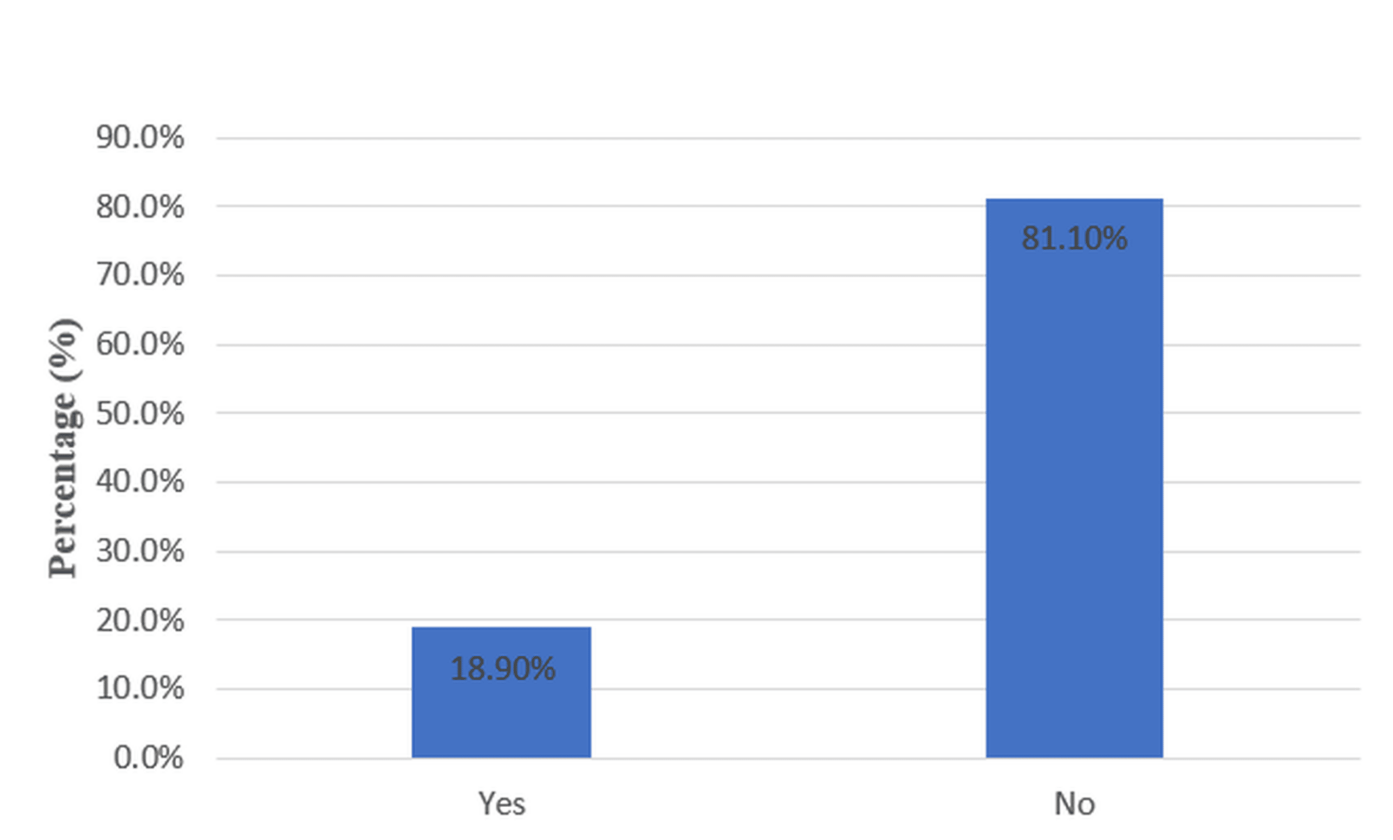
Incidence of Postoperative Acute Kidney Injury

Among 175 patients, postoperative AKI was noted in 33 (18.9%) patients, while 142 (81.1%) patients did not develop AKI (Figure 1).

Patients with AKI were significantly older (61.85 ± 8.51 vs. 56.23 ± 11.33 years; p = 0.008) and had higher baseline serum/Cr (1.12 ± 0.25 vs. 0.99 ± 0.15 mg/dL; p < 0.001). Significant predisposing factors included hypertension (OR = 6.52, p < 0.001), DM (OR = 3.11, p=0.004), ischemic heart disease (OR =3.47, p =0.010), low preoperative hemoglobin <11 mg/dL (OR = 5.13, p < 0.001), and exposure to contrast agents (OR = 5.36, p = 0.001) or nephrotoxic drugs (OR = 5.74, p < 0.001). Intraoperative risk factors significantly associated with AKI included surgery duration >2 hours (OR = 6.80, p < 0.001), blood loss >300 mL (OR = 5.80, p < 0.001), and blood transfusion (OR = 4.02, p = 0.001) (Table 1).

**Table 1:** Comparative Analysis of Demographic, Clinical, and Perioperative Factors Between Patients with and without Postoperative Acute Kidney Injury Continuous variables are presented as mean ± SD and were analyzed by an independent t-test. Categorical variables are presented as number and percentage and were analyzed by Pearson’s chi-square (χ²) test. The effect size is reported as the mean difference for numerical variables and OR with 95% CI for categorical variables. AKI: Acute Kidney Injury; ASA: American Society of Anesthesiologists; CI: Confidence Interval; OR: Odds Ratio; SD: Standard Deviation.

Variable	Category	AKI (n = 33)	No AKI (n = 142)	χ² / t-value	Effect Size (OR / Mean Diff., 95% CI)	p-value
Age (years)	Mean ± SD	61.85 ± 8.51	56.23 ± 11.33	t = 2.679	5.62 (1.48–9.77)	0.008
Body Mass Index (kg/m²)	Mean ± SD	28.91 ± 3.14	28.11 ± 3.71	t = 1.151	0.80 (–0.57–2.18)	0.251
Baseline Creatinine (mg/dL)	Mean ± SD	1.12 ± 0.25	0.99 ± 0.15	t = 3.847	1.30 (0.06–1.97)	<0.001
Gender	Male	15 (45.5%)	86 (60.6%)	χ² = 2.505	OR = 1.84 (0.86–3.95)	0.114
	Female	18 (54.5%)	56 (39.4%)			
Hypertension	Yes	25 (75.8%)	46 (32.4%)	χ² = 20.883	OR = 6.52 (2.73–15.57)	<0.001
Diabetes Mellitus	Yes	15 (45.5%)	30 (21.1%)	χ² = 8.296	OR = 3.11 (1.41–6.89)	0.004
Ischemic Heart Disease	Yes	8 (24.2%)	12 (8.5%)	χ² = 6.597	OR = 3.47 (1.29–9.35)	0.010
Type of Anesthesia	General	27 (81.8%)	102 (71.8%)	χ² = 1.378	OR = 1.77 (0.68–4.60)	0.240
	Regional	6 (18.2%)	40 (28.2%)			
Hemoglobin Status	< 11 mg/dL	16 (48.5%)	22 (15.5%)	χ² = 17.146	OR = 5.13 (2.26–11.66)	<0.001
	≥ 11 mg/dL	17 (51.5%)	120 (84.5%)			
ASA Physical Status	I	3 (9.1%)	12 (8.5%)	χ² = 1.197	—	0.754
	II	10 (30.3%)	57 (40.1%)			
	III	10 (30.3%)	39 (27.5%)			
	IV	10 (30.3%)	34 (23.9%)			
Preoperative Contrast Use	Yes	8 (24.2%)	8 (5.6%)	χ² = 11.162	OR = 5.36 (1.84–15.61)	0.001
Preop. Nephrotoxic Drug Use	Yes	16 (48.5%)	20 (14.1%)	χ² = 19.393	OR = 5.74 (2.50–13.17)	<0.001
Duration of Surgery	≤ 2 hours	9 (27.3%)	102 (71.8%)	χ² = 22.919	OR = 6.80 (2.91–15.89)	<0.001
	> 2 hours	24 (72.7%)	40 (28.2%)			
Blood Loss	≤ 300 mL	19 (57.6%)	126 (88.7%)	χ² = 18.300	OR = 5.80 (2.45–13.77)	<0.001
	> 300 mL	14 (42.4%)	16 (11.3%)			
Blood Transfusion	Yes	14 (42.4%)	22 (15.5%)	χ² = 11.886	OR = 4.02 (1.76–9.19)	0.001
Type of Surgery	Laparotomy	14 (42.4%)	48 (33.8%)	χ² = 2.221	—	0.695
	Intestinal	6 (18.2%)	26 (18.3%)			
	Hepatobiliary	4 (12.1%)	20 (14.1%)			
	Pancreatic	4 (12.1%)	12 (8.5%)			
	Minor Surgery	5 (15.2%)	36 (25.4%)			

**Figure 2:**
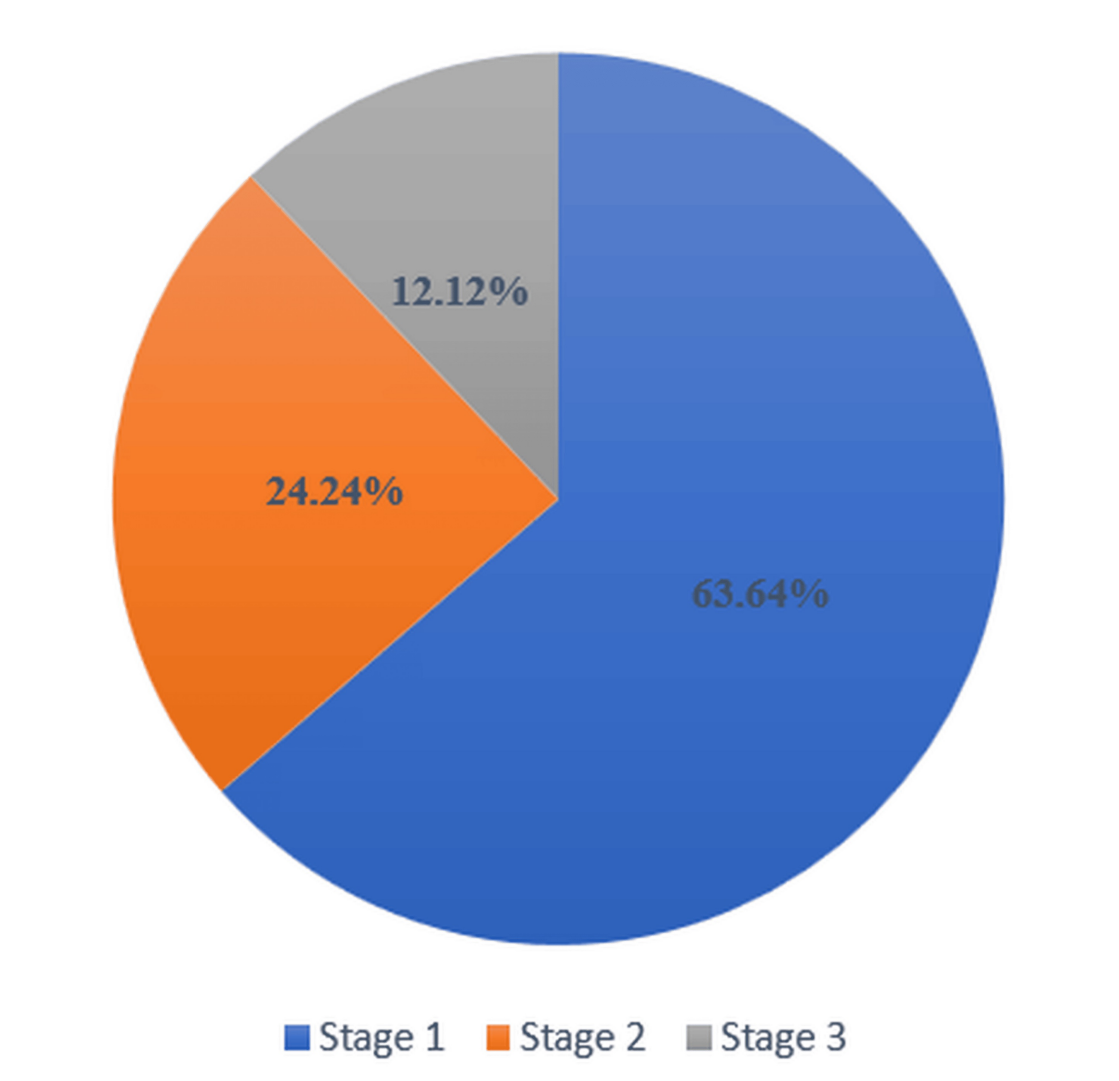
Severity of Acute Kidney Injury

Among patients who experienced postoperative AKI, 21 patients (63.64%) were grouped as Stage 1, eight patients (24.24%) as Stage 2, and four patients (12.12%) as Stage 3, based on severity grading (Figure 2).

Among patients who developed postoperative AKI, 45.5% achieved complete recovery, 36.4% had partial recovery, and 18.2% showed no recovery. ICU admission was more common in the AKI group (45.5% vs. 8.5%; p < 0.001). RRT was indicated in 18.2% of AKI patients. In-hospital mortality was also higher in the AKI patients (18.2% vs. 5.6%; p = 0.017) (Table 2).

**Table 2:** Comparison of Clinical Outcomes Between Patients with and without Postoperative Acute Kidney Injury This table presents the association of key clinical outcomes among patients with and without postoperative AKI using the chi-square test. AKI: Acute Kidney Injury; ICU: Intensive Care Unit.

Clinical Outcome Variable	AKI (n = 33)	No AKI (n = 142)	p-value
Recovery Status		-	-
Complete Recovery	15 (45.5%)		
Partial Recovery	12 (36.4%)		
No Recovery	6 (18.2%)		
ICU Admission	15 (45.5%)	12 (8.5%)	<0.001
Renal Replacement Therapy	6 (18.2%)	0 (0.0%)	<0.001
In-Hospital Mortality	6 (18.2%)	8 (5.6%)	0.017

Multivariable logistic regression identified increasing age (OR: 1.141, 95% CI: 1.009-1.291; p = 0.035), hypertension (OR: 0.001, 95% CI: <0.001-0.079; p = 0.002), and ischemic heart disease (OR: 0.047, 95% CI: 0.003-0.779; p = 0.033) as significant independent predictors of postoperative AKI. Additional risk factors included preoperative contrast use (OR: <0.001, 95% CI: <0.001-0.034; p = 0.002), nephrotoxic drug exposure (OR: 0.030, 95% CI: 0.001-0.661; p = 0.026), surgical duration >2 hours (OR: 0.010, 95% CI: <0.001-0.267; p = 0.006), and blood loss >300 mL (OR: 0.001, 95% CI: <0.001-0.106; p = 0.004) (Table 3).

**Table 3:** Multivariable Logistic Regression Analysis for Independent Predictors of Postoperative Acute Kidney Injury The table shows the results of multivariable binary logistic regression identifying independent predictors of postoperative AKI. The model revealed that increasing age, hypertension, ischemic heart disease, exposure to contrast agents, nephrotoxic drug use, longer surgical duration, and excessive intraoperative blood loss were statistically significant predictors. B: Regression Coefficient; Wald: Wald Chi-Square Test Statistic; OR: Odds Ratio; CI: Confidence Interval.

Variable	B	Wald	Adjusted OR	95% CI for OR	p-value
Age (years)	0.132	4.442	1.141	1.009 – 1.291	0.035
Hypertension (Yes vs. No)	-6.890	9.655	0.001	<0.001 – 0.079	0.002
Diabetes Mellitus (Yes vs. No)	-2.415	2.763	0.089	0.005 – 1.541	0.096
Ischemic Heart Disease (Yes vs. No)	-3.049	4.559	0.047	0.003 – 0.779	0.033
Preoperative Contrast Use (Yes vs. No)	-8.852	10.019	<0.001	<0.001 – 0.034	0.002
Type of Anesthesia (General vs. Regional)	-0.552	0.133	0.576	0.030 – 11.127	0.715
Nephrotoxic Drug Use (Yes vs. No)	-3.501	4.943	0.030	0.001 – 0.661	0.026
Duration of Surgery (>2 hrs vs. ≤2 hrs)	-4.561	7.603	0.010	<0.001 – 0.267	0.006
Hemoglobin <11 mg/dL (Yes vs. No)	-3.198	3.138	0.041	0.001 – 1.405	0.076
Blood Loss >300 mL (Yes vs. No)	-7.053	8.248	0.001	<0.001 – 0.106	0.004
Blood Transfusion (Yes vs. No)	-2.493	2.802	0.083	0.004 – 1.531	0.094

## Discussion

The incidence of postoperative AKI in this study was 18.9%, positioning it in the higher range of reported frequencies. Prior studies have shown wide variability, ranging from 2.0% to over 20%, likely reflecting differences in population characteristics, surgical complexity, perioperative practices, and AKI definitions [11,17]. Lower incidence rates were noted in some studies (e.g., 2.0% and 5.0%), while others more closely aligned with the present findings (e.g., 12.8%, 14.4%, 20.3%) [17-19]. These variations underscore the context-specific nature of AKI risk. The severity distribution in this study, 63.6% Stage 1, 24.2% Stage 2, and 12.1% Stage 3, is also consistent with previously reported data, where Stage 1 often predominates, indicating a majority of cases are initially mild but still carry clinical significance [11,13].

Advanced age was significantly associated with postoperative AKI, with affected patients being older by a mean of 5.6 years. This trend is well-supported by prior studies that implicate aging-related decline in renal reserve and autoregulatory efficiency as contributors to increased vulnerability [20]. Gender distribution showed more female patients developing AKI (54.5%), though not statistically significant. Comorbid conditions strongly influenced AKI risk. Hypertension, present in 75.8% of AKI patients, emerged as a significant risk factor. This aligns with evidence indicating impaired renal perfusion and altered autoregulation in chronically hypertensive individuals. Similarly, diabetes mellitus (45.5% in AKI vs. 21.1% in non-AKI) and ischemic heart disease (24.2% vs. 8.5%) were significantly associated with AKI, consistent with previous reports linking metabolic and cardiovascular comorbidities to perioperative renal injury [21,22]. Higher baseline serum creatinine among AKI patients (1.12 ± 0.25 vs. 0.99 ± 0.15 mg/dL) further supports the role of subclinical renal dysfunction in increasing susceptibility. Preoperative anemia (Hb <11 mg/dL) was also more common in AKI patients, reinforcing the association between oxygen delivery deficits and renal hypoperfusion [11].

Intraoperative variables demonstrated clear associations. Surgical duration exceeding two hours was significantly more common among AKI cases (72.7% vs. 28.2%), suggesting prolonged exposure to anesthetic agents, hypotension, and fluid imbalance as key contributors. Blood loss >300 mL (42.4% vs. 11.3%) and intraoperative transfusion (42.4% vs. 15.5%) were also associated with AKI, consistent with literature that links volume depletion and transfusion-related renal inflammation to postoperative renal dysfunction [16,19]. Additionally, preoperative exposure to nephrotoxic medications and contrast agents was significantly higher among AKI patients. These findings reaffirm the role of iatrogenic renal insults in AKI pathogenesis and align with established mechanisms of contrast-induced nephropathy and drug-related tubular injury [13,22]. In the present study, contrast exposure was classified as a binary variable, with no further stratification by dose, type, or number of exposures, which limits the ability to assess dose-dependent risk.

The clinical outcomes associated with AKI were notable. While 45.5% of AKI cases achieved full renal recovery, 36.4% had partial and 18.2% had no recovery at discharge. These outcomes mirror trends reported in other surgical cohorts, where residual renal dysfunction persists in a significant proportion of patients [19]. The need for ICU admission was higher in AKI patients (45.5% vs. 8.5%), reflecting the heightened care burden and complexity associated with AKI. RRT was needed in 18.2% of AKI cases, consistent with published rates of 11-19% in comparable studies [15,22]. Furthermore, in-hospital mortality among AKI patients was over threefold higher than in those without AKI (18.2% vs. 5.6%), underlining the prognostic significance of postoperative AKI and its impact on short-term surgical outcomes [17,18].

Multivariable logistic regression identified several independent predictors of postoperative AKI. Increasing age remained a consistent risk factor (OR: 1.141), reiterating the influence of age-related decline in renal compensatory mechanisms. Hypertension (OR: 0.001) and ischemic heart disease (OR: 0.047) emerged as strong predictors, likely due to chronic alterations in renal perfusion and increased hemodynamic fragility. These associations are supported by studies across surgical populations, which emphasize the cumulative burden of cardiovascular comorbidities on renal outcomes [11,22].

Iodinated contrast exposure (OR: <0.001) and nephrotoxic medications (OR: 0.030) independently predicted AKI, consistent with literature on perioperative nephrotoxicity. Among intraoperative variables, surgical duration >2 hours (OR: 0.010) and blood loss >300 mL (OR: 0.001) also retained independent significance, reflecting the role of surgical complexity and hemodynamic compromise. Notably, although diabetes mellitus, anemia, and blood transfusion were significant in univariate analysis, they lost significance in the adjusted model, likely due to mediation by other variables such as operative duration and cardiovascular status. These patterns are consistent with multivariate findings in previous large-scale analyses [18,21].

The strengths of this study include its prospective design, comprehensive assessment of perioperative variables, and robust statistical modeling through multivariable logistic regression to identify independent predictors of postoperative AKI. The inclusion of a diverse surgical population enhances the external validity within tertiary care settings. However, several limitations must be acknowledged. The single-center nature of the study may restrict generalizability, and a relatively small sample size may have limited the power to detect associations with less prevalent variables. Intraoperative hemodynamic data, fluid balance, and urine output were not recorded, potentially introducing unmeasured confounding. Additionally, the study focused only on short-term outcomes without long-term renal follow-up. Several odds ratios from the multivariable analysis were extremely low with wide confidence intervals, likely due to sparse data and limited AKI events in some groups. These associations should be interpreted with caution, and larger studies are needed to validate these findings. Future research should involve multicenter prospective studies with larger cohorts, incorporate perioperative physiological monitoring, and evaluate long-term renal and survival outcomes. Development of predictive models based on these findings could guide early identification and intervention in high-risk surgical patients.

## Conclusions

This study highlights the considerable burden of postoperative AKI among surgical patients and emphasizes its association with multiple preoperative, intraoperative, and pharmacologic risk factors. Advanced age, cardiovascular comorbidities, and prolonged surgical procedures significantly influenced the likelihood of AKI development. The condition was linked to unfavorable clinical outcomes, including reduced renal recovery, increased ICU admissions, dialysis requirement, and mortality. These findings underline the need for vigilant perioperative assessment and risk stratification. Early identification of vulnerable patients and minimization of modifiable exposures may serve as essential strategies to prevent AKI and improve postsurgical prognosis.

## Appendices

Data collection proforma

https://docs.google.com/document/d/1FLnrDex-x5NTr84Qvt9RQmwRuOU3sXK0/edit?usp=drive_link&ouid=116994616426905760969&rtpof=true&sd=true
